# The effect of alfalfa cultivation on improving physicochemical properties soil microorganisms community structure of grey desert soil

**DOI:** 10.1038/s41598-023-41005-8

**Published:** 2023-08-23

**Authors:** Jiangjiao Qi, Dongqing Fu, Xuzhe Wang, Fanfan Zhang, Chunhui Ma

**Affiliations:** College of Animal Science & Technology, Shihezi University, Shihezi, 832003 Xinjiang China

**Keywords:** Biogeochemistry, Environmental sciences

## Abstract

Planting alfalfa in grey desert soil can have significant effects on soil nutrient levels, microbial communities, and overall soil improvement. High-throughput sequencing technology was used to explore the relationship between the rhizosphere microbial community structure of grey desert soil planted with different alfalfa varieties (Aohan, WL525HQ, Knight2, Kangsai, Victoria, and WL712), alfalfa characteristics and rhizosphere soil physicochemical properties. Alfalfa planting increased the nitrogen and organic matter in the grey desert soil, and the effects in Victoria, Kangsai, and Aohan were relatively better than those in the unplanted areas and other alfalfa areas. The Chao1 and Shannon indexes showed that the diversity and relative abundance of bacteria and fungi in Kangsai were significantly higher than those in the unplanted areas and other alfalfa areas. Redundancy analysis showed that available nitrogen and phosphorus, as well as fresh weight, significantly affected the changes in fungal and bacterial communities. Variance partitioning analysis showed that soil and alfalfa growth characteristics explained 50.04% and 51.58% of the structural changes in the bacteria and fungi, respectively. Therefore, planting alfalfa changed the community structure of bacteria and fungi, as well as the content of soil nutrients, and different varieties of alfalfa had different effects on soil improvement.

## Introduction

Grey desert soil is a slightly moist gypsum-salt layer that develops on the fine soil material at the edges of temperate deserts^1^. It is distributed in the southern Junggar Basin, northern Tianshan Mountains in Xinjiang, and Hexi Corridor in Gansu, China. The gypsum content of grey desert soil in the northern Tianshan Mountains in Xinjiang is between 2 and 8%^2^. The total area of grey desert soil in Xinjiang is 1.7895 × 10^6^ ha, of which cultivated land accounts for 5.75 × 10^5^ ha; the area of effective cultivated land accounts for 32%, and the salt content is between 0.5 and 2%. Xinjiang is part of a slightly to moderately saline region, which seriously affects or restricts crop growth^3^.

Alfalfa possesses a developed root system, strong regeneration ability, and perennial nature^4^. Its potential application for improving soil environment is promising due to its ability for heavy metal enrichment, windproofing, and sand-fixing^5^. With the increasing need and awareness of environmental protection, the ecological benefits of alfalfa planting have garnered attention^6^. Several research findings support this notion, including its ability to reduce the content of high-molecular-weight polycyclic aromatic hydrocarbons in coal mining areas soils^7^, its potential as a remediation material for Cd polluted soil^8^, its efficacy in soil remediation in oil-mining areas^9^, and its ability to enhance soil structure and productivity in saline-alkali lands through continuous cropping^10^. In addition, alfalfa has been divided into different dormancy levels according to the difference of growth period and cold resistance. The dormancy characteristics of alfalfa have a crucial influence on its productivity and adaptability (e.g., wintering ability and cold resistance), and the cold resistance of low-dormancy alfalfa is higher than that of non-dormant alfalfa^11,12^. Considering soil improvement and ecological restoration, soil microorganisms have become a focal point of research and play a crucial role as a medium for alfalfa’s effectiveness.

Soil microorganisms play a vital role in the soil ecosystem, and their diversity is closely linked to soil nutrients and overall soil health^13^. Their abundance reflects and determines the ecological characteristics of the soil^14^. These microorganisms are involved in organic matter, degradation, humus formation^15^, and the transformation and cycling of soil nutrients, making them essential for improving soil structure^16^. Therefore, understanding the diversity and community composition of soil microorganisms is relevant. Soil parent materials and nutrients are the primary factors influencing soil microbial communities^17^. The rhizosphere, which refers to the thin layer of soil surrounding the root system, is a critical zone for interactions between microorganisms and plants^18^. Research has demonstrated that AMF in alfalfa symbionts can enhance plant nutrient status and improve soil conditions^19^. The rhizosphere microbial community exhibits high diversity and plays significant role in increasing nutrient availability to plant roots^20^. Rhizosphere microorganisms release metabolize acids that dissolve insoluble minerals, which plants can then absorb through their roots^21^. They also secrete indoleacetic acid to stimulate plant growth^22^. Studies have indicated that environmental changes can disrupt the balance of normal flora, leading to reduced efficient utilization of soil nutrients^23^. Apart from soil physicochemical characteristics, the microbial structure of the rhizosphere is influenced by plant strains and physiological conditions^24^. For instance, studies have observed significant variations in the soil microbial structure in rubber tree plantations over the years^25^. However, the impact of different alfalfa varieties on the rhizosphere soil flora structure in grey desert areas remains unclear.

In this study, the microbial community structure of alfalfa rhizosphere soil in a grey desert area was comprehensively analyzed and compared using high-throughput sequencing technology (partial bacterial 16s rRNA and fungal ITS genes). Our primary objective was to investigate the impact of different alfalfa varieties on the nutrient content and spatial distribution of microbial communities in grey desert soil. By doing so, we aimed to elucidate relationships between alfalfa traits, microorganisms, and soil physicochemical properties. Ultimately, our findings aim to provide valuable insights and guidance for maintaining the sustainable development of grey desert soil ecosystems.

## Results

### Soil properties

The soil in the control group (CK) of the experimental area was weakly alkaline (pH 7.26). The pH of the rhizosphere in the alfalfa area decreased significantly (*p* < 0.05), and the Kangsai soil had the lowest pH. The contents of soil organic matter (SOM), available nitrogen (AN), and total nitrogen (TN) in the rhizosphere soil of the different alfalfa varieties were significantly higher than that of CK (*p* < 0.05). Total potassium (TK) and available potassium (AK) in the Victoria and Kangsai areas were significantly lower than that of CK (*p* < 0.05). Total phosphorus (TP) and available phosphorus (AP) in the Kangsai area were higher than that of CK (*p* < 0.05). The TN and AN contents in the Kangsai and Aohan areas were significantly higher than those in other alfalfa areas. The SOM content in the Victoria and Kangsai areas was significantly higher than that the other alfal areas (*p* < 0.05) (Table 1).

**Table 1:** Soil physicochemical properties of different alfalfa varieties.

Index	Control group (CK)	WL 712	WL 525HQ	Victoria	Kangsai	Knight 2	Aohan	*p*-Values	SEM
(TN, mg/Kg)	106.48 ± 0.33^f^	118.50 ± 0.87^e^	115.57 ± 0.18^e^	135.57 ± 0.18^c^	150.19 ± 0.47^a^	121.20 ± 2.10^d^	137.60 ± 0.95^b^	0.000	0.193
(AN, mg/Kg)	92.50 ± 0.27^d^	95.32 ± 0.63^c^	95.65 ± 0.13^c^	94.63 ± 0.28^c^	108.98 ± 0.47^a^	95.43 ± 0.91^c^	97.20 ± 0.28^b^	0.000	0.108
(TP, g/Kg)	0.62 ± 0.01^c^	0.66 ± 0.01^a^	0.52 ± 0.01^e^	0.63 ± 0.01^b^	0.66 ± 0.01^a^	0.59 ± 0.01^d^	0.58 ± 0.01^d^	0.000	0.001
(AP, mg/Kg)	33.34 ± 0.35^b^	34.43 ± 0.06^a^	28.59 ± 0.74^d^	31.77 ± 0.50^c^	33.42 ± 0.43^b^	29.03 ± 0.60^d^	25.57 ± 0.38^e^	0.000	0.098
(TK, g/Kg)	23.60 ± 0.61^a^	22.33 ± 0.75^a^	19.33 ± 1.07^b^	18.53 ± 0.81^bc^	17.20 ± 0.56^c^	18.47 ± 0.97^bc^	19.90 ± 0.92^b^	0.000	0.171
(AK, mg/Kg)	168.54 ± 0.27^d^	209.47 ± 0.90^a^	156.21 ± 0.99^e^	149.60 ± 0.24^f^	139.48 ± 0.41^g^	175.97 ± 0.58^c^	185.57 ± 0.15^b^	0.000	0.120
(SOM, g/Kg)	12.32 ± 0.26^f^	34.90 ± 0.79^e^	36.87 ± 0.55^d^	41.67 ± 0.25^a^	40.50 ± 1.25^b^	37.57 ± 0.58^cd^	38.23 ± 1.13^c^	0.000	0.137
PH	7.26 ± 0.03^a^	6.50 ± 0.18b^bc^	6.53 ± 0.02^b^	6.63 ± 0.01^b^	6.33 ± 0.04^c^	6.70 ± 0.13^b^	6.52 ± 0.19^bc^	0.000	0.023

Different letters in the same row indicate significant differences among different alfalfa varieties. Significant values are denoted as follows: different small letters, *p* < 0.05. Data are presented as the mean ± standard error.

### Phenotypic analysis of six alfalfa varieties

The stem diameter (SD) of WL712 and Kangsai was significantly higher than that of the other alfalfa varieties (*p* < 0.05). The plant height (PHT) of Victoria, Kangsai, and Aohan was significantly lower than that of WL712 (*p* < 0.05). The internode length (ILH) of WL712 and WL525HQ was significantly higher than that of the other alfalfa varieties (*p* < 0.05). The number of branches (NOBs) of Kangsai, Knight2, and Aohan were significantly higher than those of WL712 and WL525HQ (*p* < 0.05). The fresh weight (FW) of Kangsai was significantly higher than that of the other alfalfa varieties (*p* < 0.05) (Table 2).

**Table 2:** Agronomic characteristics of different alfalfa varieties.

Index	WL 712	WL 525HQ	Victoria	Kangsai	Knight 2	Aohan	*p*-Values	SEM
SD (mm)	3.57 ± 0.10^a^	3.45 ± 0.06^ab^	3.38 ± 0.03^bc^	3.56 ± 0.10^a^	3.29 ± 0.09^c^	3.36 ± 0.08^bc^	0.009	0.020
PHT (cm)	75.47 ± 1.25^a^	74.77 ± 1.46^a^	70.93 ± 1.60^b^	71.83 ± 1.45^b^	67.56 ± 1.56^c^	71.60 ± 1.30^b^	0.000	0.349
NOB (Pieces)	42.7 ± 1.53^c^	41.3 ± 1.53^c^	46.3 ± 3.05^b^	57.3 ± 0.58^a^	59.7 ± 2.52^a^	57.3 ± 2.08^a^	0.001	0.430
ILH (cm)	8.14 ± 0.06^a^	8.17 ± 0.12^a^	7.38 ± 0.04^bc^	7.46 ± 0.05^b^	7.31 ± 0.09^bc^	7.24 ± 0.12^c^	0.000	0.020
FW (g/ plant)	277.24 ± 2.08^b^	255.72 ± 1.84^c^	231.46 ± 2.21^d^	294.83 ± 3.21^a^	227.42 ± 1.68^e^	258.64 ± 1.80^c^	0.000	0.522

Different letters in the same row indicate significant differences among different alfalfa varieties. Significant values are denoted as follows: different small letters, *p* < 0.05. Data are presented as the mean ± standard error.

PHT, plant height; SD, stem diameter; FW, fresh weight; ILH, Internode Length; NOB, Number of Branches.

### Sequencing analysis

The Shannon–Wiener curve showed that the diversity analysis dataset was sufficiently large to reflect the diversity of bacteria and fungi in the sample (Fig. S1A, B). The end of the rarefaction curve tended to saturate gradually, indicating that the sequencing data reasonably covered microbial diversity (Fig. S1C, D). Coverage reflects the coverage of the microbial community. Table 3 shows that the coverage was ≥ 97%, which can represent the real situation of microorganisms in the sample, indicating that most of the types of bacteria and fungi were detected in the sample.

**Table 3:** Diversity index of bacterial and fungal communities in the rhizosphere soil of six alfalfa varieties.

		CK	WL 712	WL 525HQ	Victoria	Kangsai	Knight2	Aohan	*p*-Value	SEM
Bacterial	Chao 1	3437.30 ± 14.61^f^	3467.42 ± 9.33^e^	3356.90 ± 14.34^g^	3701.38 ± 9.82^b^	3969.77 ± 13.61^a^	3635.97 ± 9.65^c^	3592.09 ± 15.96^d^	0.000	2.931
	Shannon	6.34 ± 0.01^b^	6.35 ± 0.08^b^	6.19 ± 0.12^c^	6.43 ± 0.04^ab^	6.51 ± 0.05^a^	6.40 ± 0.02^ab^	6.37 ± 0.07^b^	0.002	0.014
	Coverage	0.99 ± 0.002^a^	0.97 ± 0.005^d^	0.97 ± 0.004^cd^	0.98 ± 0.004^bc^	0.98 ± 0.001^b^	0.98 ± 0.001^b^	0.98 ± 0.002^bc^	0.000	0.001
Fungual	Chao 1	139.48 ± 1.05^f^	172.94 ± 2.55^c^	171.47 ± 1.80^c^	149.23 ± 2.68^e^	185.83 ± 3.73^a^	180.44 ± 2.35^b^	164.64 ± 2.35^d^	0.000	0.517
	Shannon	2.68 ± 0.12^d^	3.11 ± 0.06^c^	3.34 ± 0.05^b^	3.24 ± 0.14^bc^	3.57 ± 0.05^a^	3.39 ± 0.21^ab^	3.26 ± 0.08^bc^	0.000	0.024
	Coverage	0.99 ± 0.000^a^	0.99 ± 0.000^abc^	0.99 ± 0.000^abc^	0.99 ± 0.000^ab^	0.99 ± 0.001^abc^	0.99 ± 0.000^c^	0.99 ± 0.000^bc^	0.046	0.000

Different letters in the same row indicate significant differences among different alfalfa varieties. Significant values are denoted as follows: different small letters, *p* < 0.05. Data are presented as the mean ± standard error.

We obtained 1,179,163 high-quality 16s rRNA sequences from 18 alfalfa rhizosphere samples and five CK samples, and 33,656–68,183 analysis sequences (average length of 416.9 bp) were obtained from each sample (Table S1). The bacterial OTUs were compared and annotated in the SILVA database, and the bacteria were divided into 38 phyla, 130 classes, 305 orders, 475 families and 888 genera. In total, 1,613,617 high-quality ITS sequences were obtained from 23 soil samples, and 69,194–82,152 analysis sequences (average length of 236.0 bp) were obtained from each sample (Table S2). Fungal OTUs were compared and annotated in the UNITE database, and the fungi were divided into 14 phyla, 41 classes, 85 orders, 173 families and 354 genera.

The groups common among the soils were regarded as core communities. Figure S2 shows that the number of OTUs shared by all soil bacterial and fungal samples was 2034 and 198, respectively.

### Diversity of the soil microbial community in alfalfa

Homogenization was performed with the minimum number of samples, and diversity was statistically analyzed based on the homogenized output data. The diversity and relative abundance indices of the rhizosphere microbial communities of the alfalfa varieties differed (Table 3). The Chao1 index of the bacteria was the highest in Kangsai, followed by Victoria, Knight2, Aohan, and CK; the lowest was in WL525HQ. The Chao1 index of the fungi was the highest in Kangsai, followed by WL712 and Knight2; the lowest was in CK (*p* < 0.05).

The Shannon index showed that the Kangsai bacterial diversity was significantly higher than that of CK, and the lowest was in WL525HQ (*p* < 0.05). The diversity of soil fungi in the alfalfa varieties was significantly higher than that in CK; the diversity of soil fungi in Kangsai was the highest, and the diversity of soil fungi in WL712 was the lowest (*p* < 0.05).

**Figure 1:**
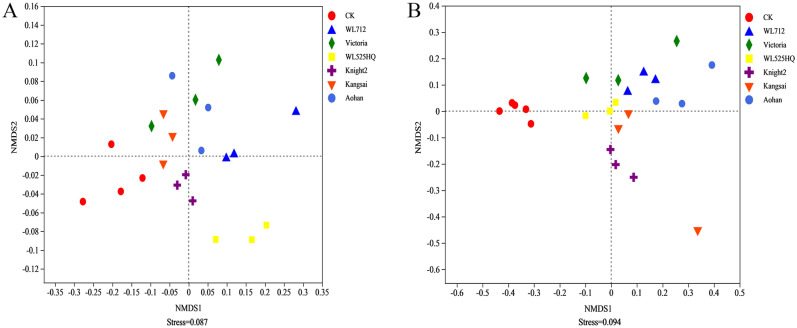
Non-metric multidimensional scaling (NMDS) analysis of bacteria (**A**) and fungi (**B**). Points with different colors or shapes represent the samples in different groups. The distance of the sample points represents the similarity of the composition of the sample species. Stress lower than 0.1 shows that NMDS analysis is representative. The closer the samples in the graph, the higher their similarity.

*β*-diversity was used to reflect the differences in microbial composition among the groups. Non-metric multidimensional scaling (NMDS) analysis showed that the bacterial communities in the Victoria, Knight2, Kangsai, and Aohan alfalfa soils were the closest. CK, WL525HQ, and WL712 had the largest distances between sequences (Fig. 1A). The different alfalfa varieties of soil fungal communities were the closest, but the distance between the CK and the rhizosphere fungal communities in the alfalfa area was large (Fig. 1B). NMDS revealed a unique structure of soil microorganisms between the alfalfa planting area and CK.

### Composition and relative abundance of core microorganisms

**Figure 2:**
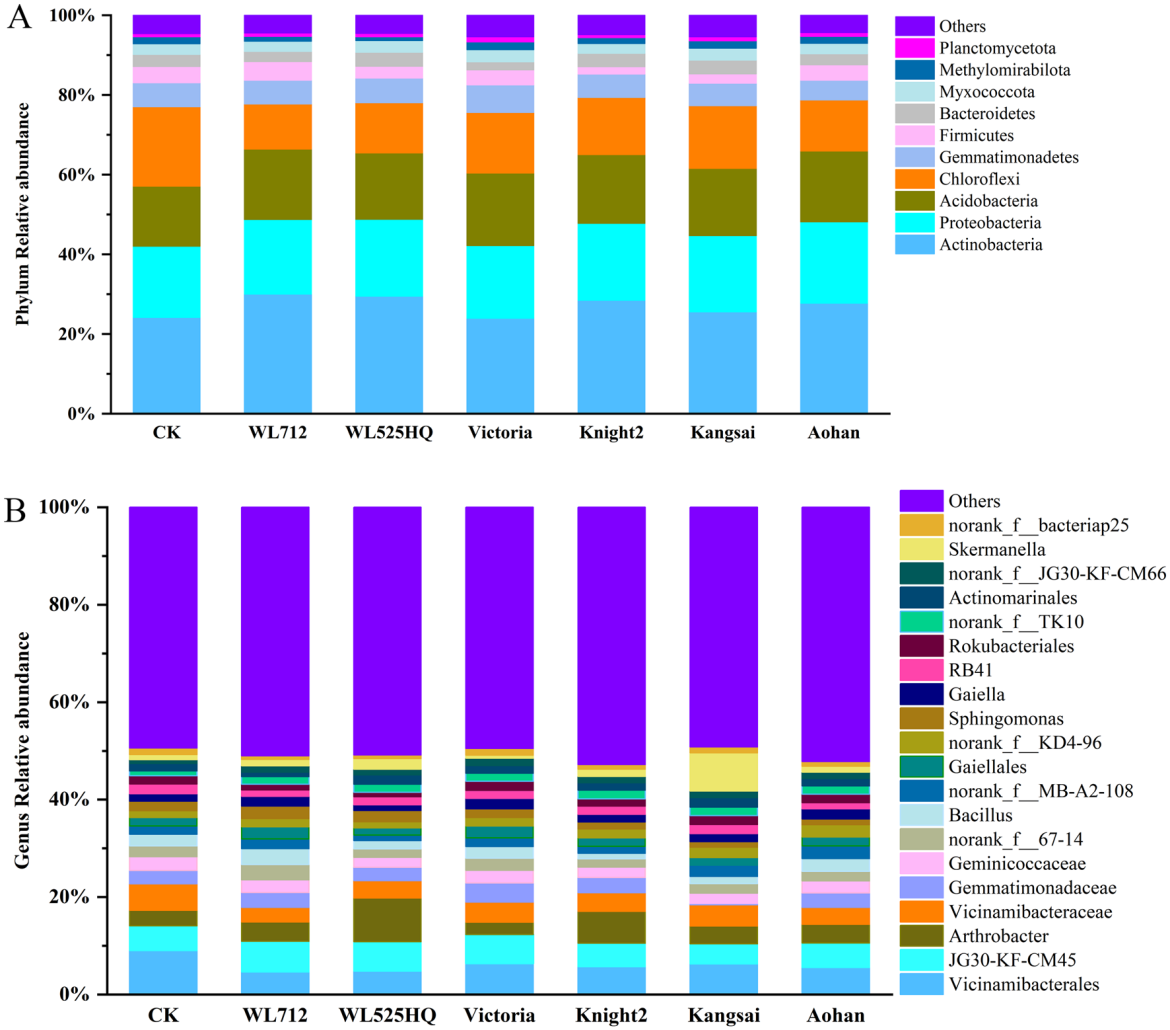
Histogram of relative abundance of bacteria. X-axis represents groups. Y-axis represents relative abundance presented as a percentage. (**A**) Relative abundance of top 10 phyla. (**B**) Relative abundance of top 20 genera.

The bacterial phyla with high relative abundances were Actinobacteria, Proteobacteria, Chloroflexi, and Acidobacteria, followed by Gemmatimonadetes and Firmicutes (Fig. 2A). More than 82% of these bacteria were detected in different groups, and the bacterial phyla with significant differences were Firmicutes and Methylomirabilota (*p* < 0.05) (Fig. 2A, Table S3). The dominant genera were *Vicinamibacterales*, *JG30-KF-CM45*, *Gemmatimonadetes*, and *Arthrobacter* among the groups (Fig. 2B). The bacterial genera with significant differences were *Arthrobacter, Bacillus, Sphingomonas, Gaiella,* and *Skermanella* (the top 20 in relative abundance) (*p* < 0.05) (Fig. 2B, Table S4).

**Figure 3:**
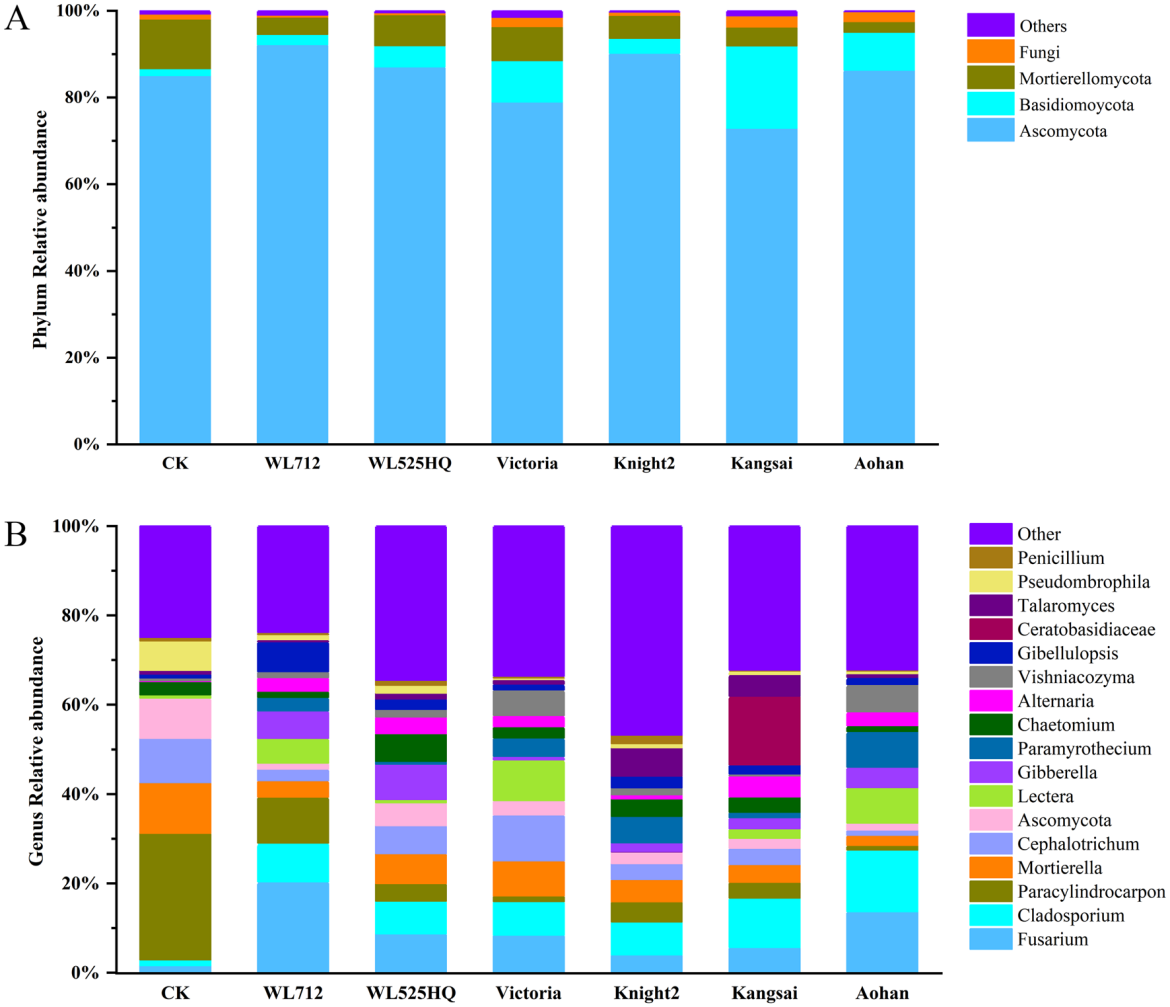
Histogram of relative abundance of fungi. X-axis represents groups. Y-axis represents relative abundance presented as a percentage. (**A**) Relative abundance of the phyla. (**B**) Relative abundance of the genera.

The fungal phyla with relatively high abundance were Ascomycota, Basidiomycota, and Mortierellomycota (Fig. 3A). More than 96% of these fungi were detected in different groups, and the fungal phyla with a significant difference was Basidiomycota (*p* < 0.05) (Fig. 3A, Table S5). Additionally, the dominant genera were *Fusarium*, *Cladosporium*, *Paracylindrocarpon*, *Mortierella*, and *Cephalotrichum* among the groups (Fig. 3B). The fungal genera with significant differences were *Fusarium*, *Lectera*, *Gibberella*, *Paracylindrocarpon*, *Cladosporium*, *Cephalotrichum*, *Alternaria*, *Gibellulopsis*, *Vishniacozyma*, *Talaromyces*, *Pseudombrophila*, and *Paramyrothecium* (Fig. 3B, Table S6).

### Factors related to the change in soil microbial community diversity

**Figure 4:**
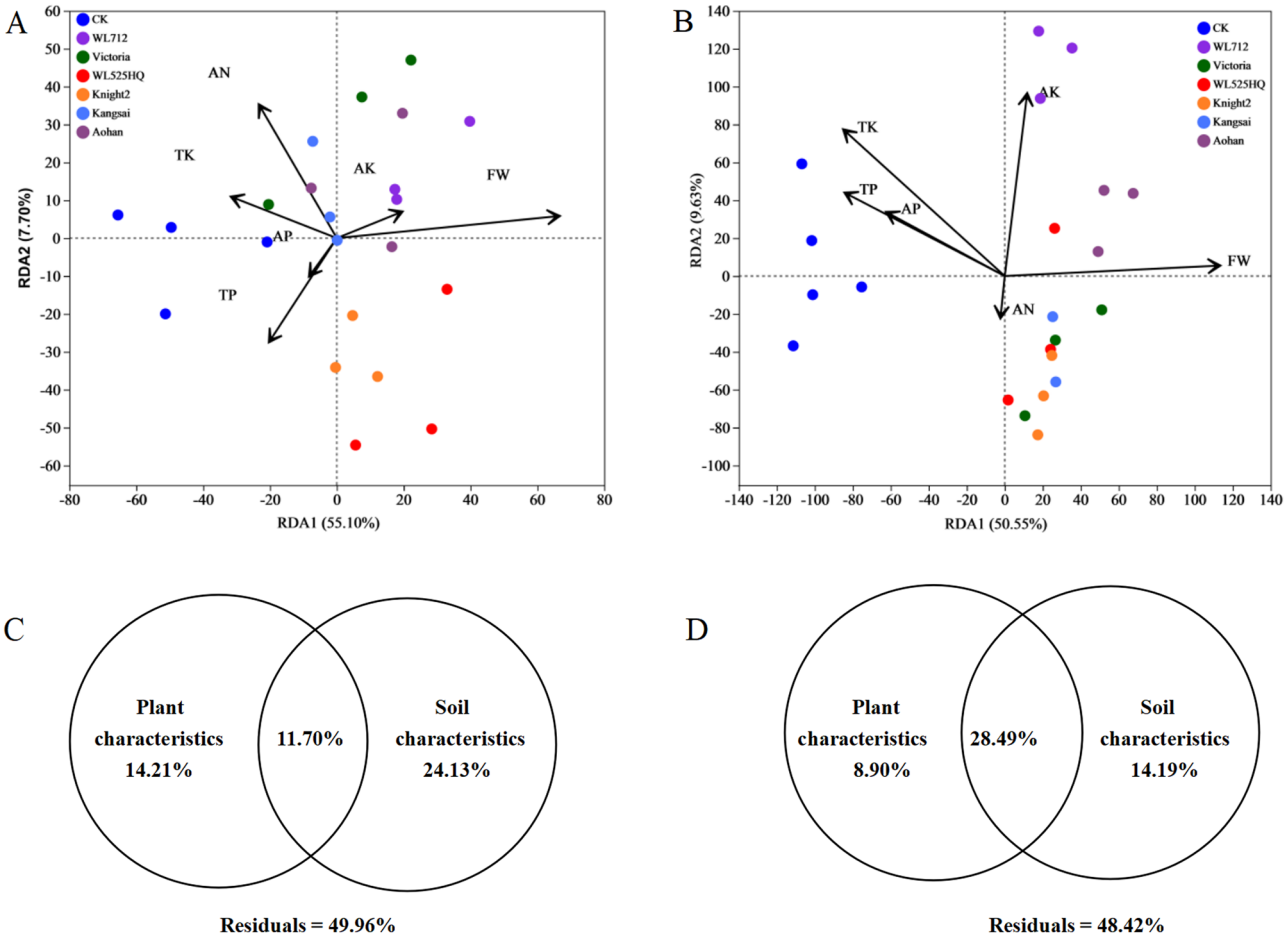
Redundancy analysis (RDA) of sequencing data (symbols) and environmental characteristics (arrows). Bacterial and fungal communities are shown in (**A** and **B**) respectively. Values of axes 1 and 2 are the percentages explained by the corresponding axis. Analysis of the level of contribution of the soil and plant characteristics to changes in bacterial (**C**) and fungal (**D**) communities.

Alfalfa planting changed the soil microbial community structure and physicochemical properties. Changes in the grey desert soil microbial community structure were mediated by differences in alfalfa growth and soil properties. RDA is mainly used to reflect the relationship between the flora and other factors. After removing of redundant variables, six influential factors were selected for RDA. AN (R^2^ = 0.2835, *p* = 0.04) and FW (R^2^ = 0.6812, *p* = 0.001) significantly affected the bacterial community structure (Fig. 4A, Table S7). TP (R^2^ = 0.6111, *p* = 0.001), AP (R^2^ = 0.3354, *p* = 0.022), TK (R^2^ = 0.8784, *p* = 0.001), AK (R^2^ = 0.5524, *p* = 0.002), and FW (R^2^ = 0.8735,* p* = 0.001) also significantly affected fungal community structure (Fig. 4B, Table S8).

Variance partition analysis (VPA) evaluates the contribution of environmental characteristics to the structures of fungi and bacteria (Fig. 4). The environmental characteristics explained 50.04% and 51.58% of bacterial and fungal structural changes, respectively (Fig. 4C, D). Alfalfa and soil characteristics explained 11.70% and 24.13% of bacterial structure changes and 8.90% and 14.19%, respectively, of fungal structure changes. Notably, the common explanations of environmental factors with respect to bacteria and fungi were 11.70% and 28.49%, respectively, which revealed the close interaction between alfalfa and the soil microbes in grey desert.

**Figure 5:**
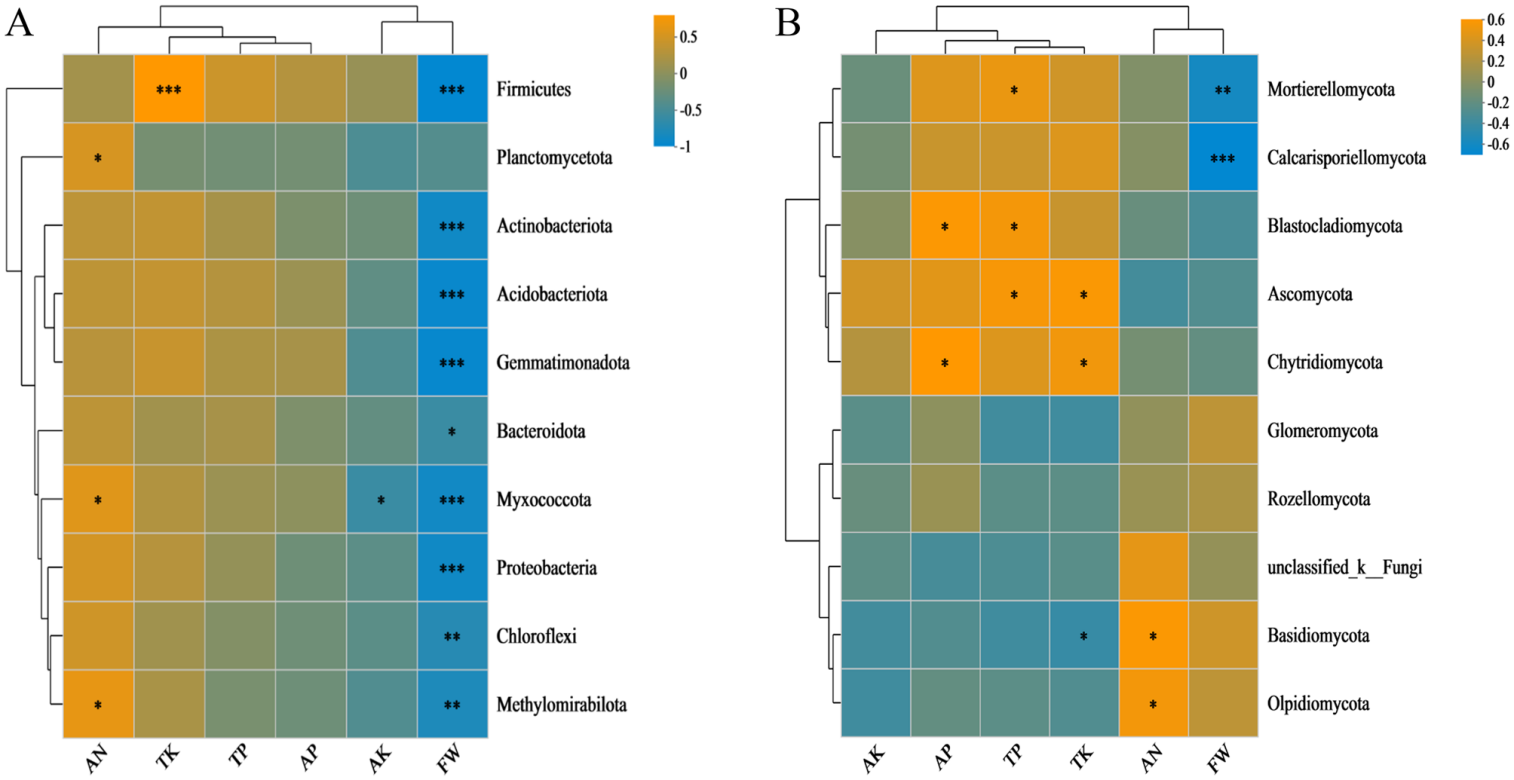
Heatmap shows relationship between bacterial (**A**) and environmental variables and relationship between fungi (**B**) and environmental variables. The X and Y axes represent environmental factors and species, respectively. R is shown in different colors. P values less than 0.05 are marked with *. * 0.01 pP < 0.05, ** 0.001 p < 0.01, or *** p < 0.001.

There was a significant correlation between the microorganisms and environmental factors at the phylum level (Fig. 5). Figure 5A shows that Firmicutes, Actinobacteria, Acidobacteria, Bacteroidetes, Gemmatimonadota, Myxococcus, Proteobacteria, Methylomirabilota, and Chloroflexi were significantly negatively correlated with FW. In addition, Firmicutes showed a significant positive correlation with TK. Myxococcus were significantly negatively correlated with AK. Planctomycetota, Myxococcota, and Methylomirabilota were significantly positively correlated with AN (Table S9). Figure 5B shows that Mortierellomycota and Calcarisporiellomycota were significantly and negatively correlated with FW. Basidiomycota and Olpidiomycota were significantly and positively correlated with AN. Mortierellomycota, Blastocladiomycota, and Ascomycota were significantly and positively correlation with TP. Chytridiomycota and Ascomycota were significantly and positively correlated with TK. Blastocladiomycota and Chytridiomycota were significantly and positively correlated with AP (Table S10).

### Analysis of significant differences among soil microbes

**Figure 6:**
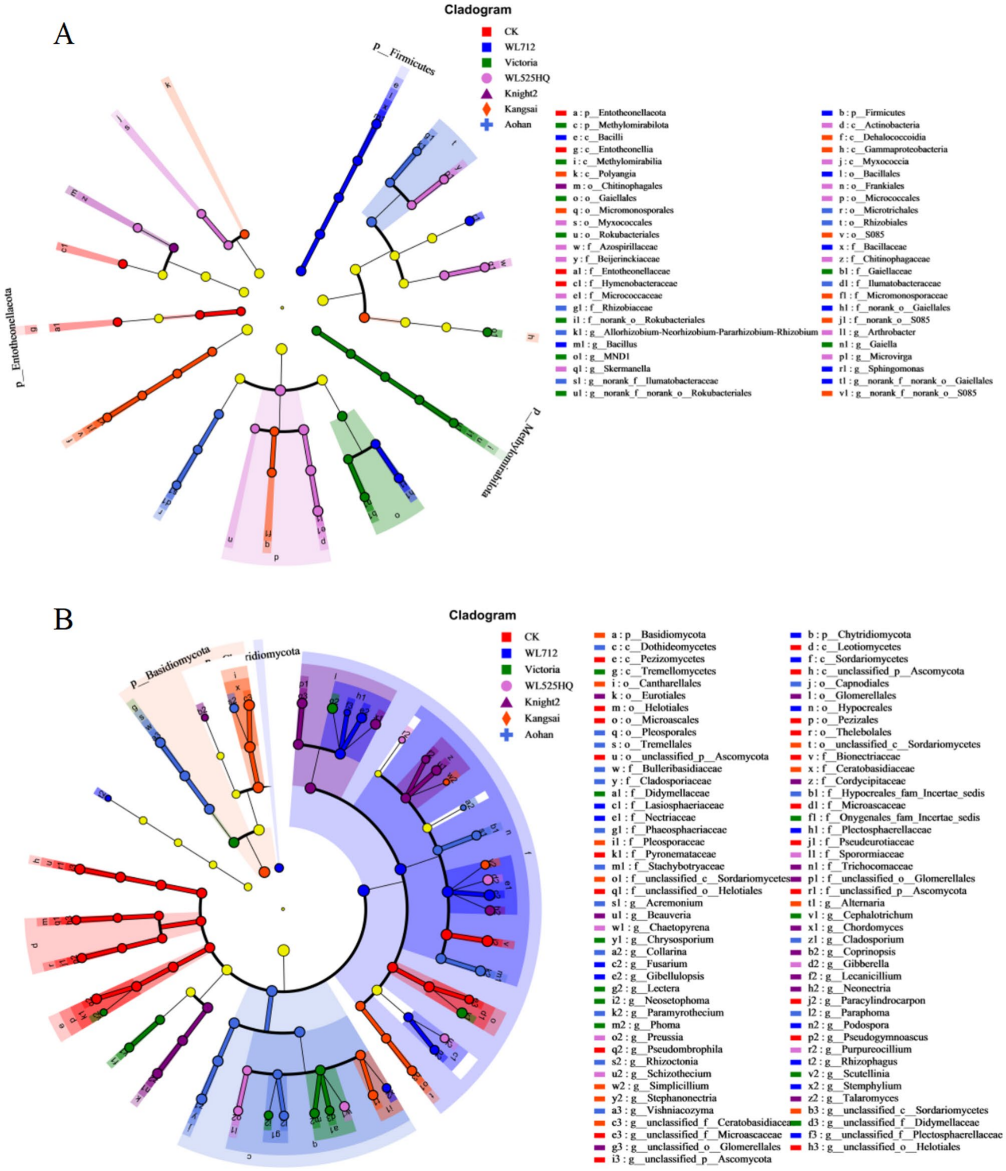
Cladogram showing phylogenetic distribution of bacterial and fungal lineages in the soil. Circles indicate phylogenetic levels from domain to genus. The diameter of each circle is proportional to the abundance of the group. Nodes with different colors represent significantly enriched microbial groups. Light yellow nodes indicate microbial groups without significance.

Identifying specific biomarkers in the samples holds greater significance than analyzing the overall abundance and diversity of microorganisms. Through LefSe analysis, we discovered 48 distinct bacterial taxa (Fig. 6A) and 87 unique fungal taxa (Fig. 6B) that exhibited significant differences among the soil samples from the experimental areas.

**Figure 7:**
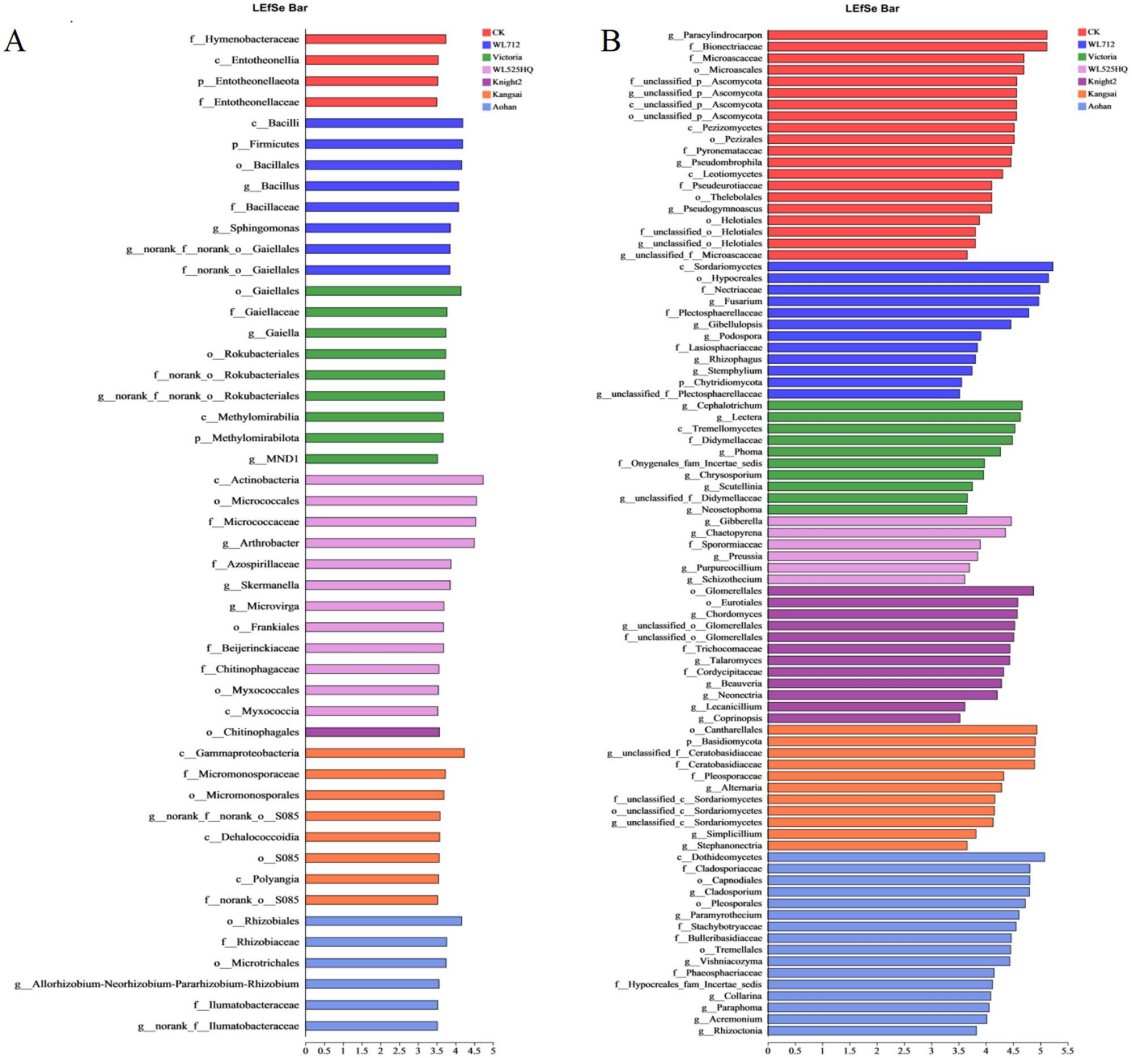
LDA of bacterial and fungal lineages in the soil. The groups of LDA ≥ 3.5 were marked as special and having significant differences. Different-colored regions represent different constituents.

Figure 7A and B show the linear discriminant analysis (LDA) scores of the bacterial and fungal communities with significant differences among the groups (Tables S11 and S12). *Sphingomonas* and *Bacillus* were significantly enriched in WL712 group. *Gaiella*, *Rokubacteriales*, and *MND1* were significantly enriched in Victoria group. *Arthrobacter*, *Skermanella*, and *Microvirga* were significantly enriched in the WL525HQ group. *Rhizobium* was significantly enriched in the Aohan samples (Fig. 7A).

At the fungal genus level, *Fusarium*, *Gibellulopsis*, *Podospora*, *Rhizophagus*, and *Stemphylium* were significantly enriched in WL712. *Cephalotrichum*, *Lectera*, *Phoma*, *Chrysosporium*, *Scutellinia*, and *Neosetopyrena* were significantly enriched in Victoria group. *Gibberella*, *Preussia*, *Purpureocillium*, *Schizothecium*, and *Chaetopyrena* were significantly enriched in the WL525HQ group. *Chordomyces*, *Glomerellales*, *Talaromyces*, *Beauveria*, *Lecanicillium*, *Coprinopsis*, and *Neonectria* were significantly enriched in the Knight2 group. *Alternaria*, *Simplicillium*, and *Stephanonectria* were significantly enriched in the Kangsai samples. *Collarina*, *Paraphoma*, *Acremonium*, *Paramyrothecium*, *Vishniacozyma*, *Rhizoctonia*, and *Cladosporium* were significantly enriched in the Aohan samples. *Paracylindrocarpon*, *Pseudogymnoascus*, and *Pseudombrophila* were significantly enriched in CK (Fig. 7B). The relative abundances of bacterial and fungal communities with significant differences among the groups are shown in Figs. S3 and S4, respectively.

## Discussion

At the experimental station, we established five control plots and 18 alfalfa plots. Among the different alfalfa varieties, Kangsai alfalfa demonstrated a significant decreased in soil pH, while its fresh weight was higher than that of the other varieties (Table 1). This finding aligns with the understanding that high-yield alfalfa varieties tend to be more tolerant to low soil pH^26^. Additionakky, the biological nitrogen fixation process in alfalfa root nodules can enhance soil nitrogen fertility^27^. Compared with the CK, alfalfa planting significantly increased the TN, AN, and SOM content of the soil (Table 1), similar to the results in the literature^28^.

An appropriate supply of nitrogen can promote the vigorous growth of crops and increase crop yield^29,30^. P and K can enhance the adaptability of crops to the external environment and the robustness of crop stalks, respectively^31^. In this study, AN and AP in WL712 and Kangsai were higher than those in CK and the other alfalfa areas, and the FW and SD of WL712 and Kangsai were significantly better than those of the other alfalfa areas (Tables 1 and 2). These results may be the result of plant-soil-microbial interactions, which is similar to results in the literature.

The Chao1 and Shannon indices revealed that the diversity and relative abundance of fungi in the alfalfa planting area were significantly higher than those in the CK, and the relative abundance and diversity of the bacteria and fungi in Kangsai were the highest (Table 3). Moreover, the TN, AN, and SOM contents in Kangsai were higher than those in the CK and other alfalfa areas. These finding are consistent with previous studies^32^, suggesting that alfalfa may improve substance circulation o and ecological structure of the soil by altering the structure of the microbial community.

The histogram shows that Actinobacteria, Proteobacteria, Gemmatimonadetes, Chloroflexi, Acidobacteria, and Firmicutes were the dominant bacterial phyla (relative abundance was higher than 1%), while Ascomycota, Basidiomycota, and Mortierellomycota were the dominant fungal phyla. Regardless of whether alfalfa was planted, the composition of the soil microbial communities was basically the same, but the relative abundances were significantly different (Figs. 2A and 3A). Typically, according to the literature, there is no difference in the diversity of microbial communities between natural and agricultural soils^33^. Thus, using alfalfa to improve the grey desert soil structure does not necessarily mean that the diversity of bacteria and fungi will decrease or disappear^34^. Bi et al.^35^ found that Proteobacteria and Acidobacteria were the main groups observed during the transformation of soil types. In this study, the relative abundances of Proteobacteria and Acidobacteria were 19.01% and 14.58%, respectively. Additionally, Acidobacteria belong to acidophilic and oligotrophic flora, which mostly grow in acidic and nutrient-deficient soils^36^. We found that the relative abundance of Acidobacteria in the alfalfa planting area increased significantly, which may be related to soil acidity. Actinobacteria grow in neutral or weakly acidic soils rich in organic matter^37^. We found that the abundance of Actinobacteria in the alfalfa cultivation area was higher than that in CK. These results may also be related to an increase in organic matter and a decrease in soil pH.

The cultivation of different alfalfa varieties contributed differently to the soil microbial communities (Fig. 4A, B). Research has reported that alfalfa cultivation changes soil microbial community structure and soil characteristics, which might be the result of interactions between plants and microorganisms^25^. Notably, the diversity of soil microorganisms may be related to the plant cultivars^38^. The *β*-diversity results were consistent with those in the literature (Fig. 1). Changes in microbial communities were closely related to soil characteristics (particularly soil N content) and plant growth^39^. The cultivation of alfalfa significantly increased the nitrogen content of the soil, which may be related to the nitrogen-fixing ability of leguminous forage (Table 1). Nitrogen is important for maintaining the life activities of plants; therefore, plant growth may consume soil nitrogen, and microbial activities may also utilize and transform soil nitrogen^40^. These results may also be one of the reasons for the difference in the nitrogen content among the different alfalfa soils (Table 1). Additionally, more than 50% of the microbial community changes were explained by the characteristics of alfalfa and soil, which indicated that environmental factors were the main factors affecting the microbial community structure. The cultivation of alfalfa improved soil structure, which was beneficial for soil nutrient circulation and carbon and nitrogen storage^27^. In this study, the cultivation of Kangsai, Victoria, and Aohan improved the grey desert soil nutrients (TN, AN, and SOM). Kangsai, Victoria, and Knight 2 alfalfa had the highest abundance and diversity of soil microbial communities (Table 3).

LefSe analysis revealed that *Bacillus* and *Sphingomonas* were special bacteria with significant differences between WL712 and the other experimental groups at the genus level (Fig. 6, Table S11). *Bacillus* spp. can produce a range of antibiotics that inhibit the propagation of harmful microorganisms and maintain soil health^41^. *Sphingomonas* reduce proline content in plant roots and weaken plant stress resistance^42^. Cold stress increases the proline content of alfalfa roots, with low dormancy^43^. We found that the relative abundance of *Sphingomonas* in WL712 was higher than that in the other areas (Fig. 2B). The interaction between alfalfa and soil microorganisms may be one reason for the good growth performance and low overwintering rate of WL712 plant. *Arthrobacter* and *Microvirga* were significantly enriched in the WL525HQ soil. In addition to denitrification, *Arthrobacter* inhibits the reproduction of pathogenic bacteria^44^. *Microvirga* ia an alkalophilic bacterium that plays a role in denitrification and reduction of nitrates^45^. These bacteria might play a role in the improvement and maintenance of grey desert soil. *Gaiella*, *Rokubacteriales*, and *MND1* were significantly enriched in Victoria soil. The abundance of the rhizosphere growth-promoting bacterium *MND1* positively correlated with crop yield and SOM content^46^. *Rokubacteriales* were significantly enriched in farmland soils polluted with heavy metals^47^. *Rhizobium* was significantly enriched in Aohan soil. *Rhizobium* fixes biological nitrogen, which can improve soil nutrients by increasing soil ammonium nitrogen in soil^48^. Our results are consistent with those in the literature (Table 1).

*Fusarium* and *Gibellulopsis* were special fungi with significant differences between WL712 and other experimental areas at the dominant genus level (Fig. 7, Table S12). Most *Fusarium* species can synthesize toxic mycotoxins that threaten the health of the soil and plants^49^. The increased abundance of *Gibellulopsis* had a certain effect on slowing own soil salinization^50^. The relative abundance of *Fusarium* in Kangsai and Knight 2 was lower than that in other alfalfa areas. *Gibberella* and *Chaetopyrena* were significantly enriched in WL525HQ soil. *Gibberella* can produce gibberellins to improve the growth and salt tolerance of crops^51^. *Cephalotrichum* and *Phoma* were significantly enriched in Victoria soil. *Cephalotrichum* is considered a potential strain for remediation as pollution in soil^52^. *Phoma* can improve the tolerance of crops^53^. *Sordariomycetes* and *Chordomyces* are strongly alkali-tolerant and effective alkalophilic bacteria^54^. *Talaromyces* is a salt-tolerant fungi fungus that produce chitinases and chitosanases^55^. The relative abundances of *Sordariomycetes*, *Chordomyces*, and *Talaromyces* in the Kangsai and Knight 2 areas were higher than those in the other areas. The relative abundance of *Cladosporium* in Kangsai and Aohan was higher than that in other areas (Fig. 3B, Table S6). *Cladosporium* effectively removed imazalil from industrial wastewater^56^. In summary, almost all significantly enriched microbial communities play a role in the improvement of grey desert soil structure and healthy plant growth.

## Conclusion

High-throughput sequencing showed that alfalfa cultivation changed the microbial community structure in grey desert soil, and Kangsai alfalfa increased the diversity and relative abundance of bacterial and fungal communities. The alfalfa varieties had different effects on soil nutrient content, and the improved effects of Victoria, Kangsai, Knight2, and Aohan were better than those of the other varieties. Additionally, Changes in the microbial community were mainly attributed to AN, AP, and FW. Considering the soil nutrients, microbial community structure, and alfalfa growth characteristics, the Kangsai alfalfa variety is more suitable for grey desert soil structure improvement. Our research provides a new direction for selecting alfalfa varieties suitable for grey desert soil improvement.

## Materials and methods

### Sample collection

The study site is located at an experimental station of Shihezi University, Xinjiang (N44.20°, E88.30°, altitude 420 m), with a temperate continental arid climate and an annual average temperature of 8.1 °C. Varieties of alfalfa seeds were donated by the Grassland Research Institute of the China Academy of Agricultural Sciences. Before seeding, the study area was plowed and harrowed without applying organic fertilizers or pesticides. During alfalfa growth, field management comprised manual weeding and timely watering (after mowing, before winter, and after returning to green).

The study area measured 150 m × 200 m. In May 2020, 18 experimental plots (alfalfa planting areas) and five control plots (cultivated soil without any vegetation, CK) were established (20 m × 30 m), and a 10 m buffer zone was established between the experimental plots. Six alfalfa varieties (Aohan, Victoria, Kangsai, Knight2, WL525HQ, and WL712) (Table 4) were planted in the experimental area, with a 0.25 m plant spacing and 0.3 m row spacing. Three experimental plots not adjacent to each other were randomly selected for each alfalfa variety. In August 2021, 50 alfalfa plants were randomly selected from each experimental site by using the “S” sampling method, the aboveground section (with a stubble of 5 cm) was mowed, and the whole root system was dug out. 50 rhizosphere soil samples were obtained by using the “root shaking” method^57^ and uniformly mixed as a biological duplication, and three biological duplicate soil samples were obtained for each alfalfa variety. The soil sampling method for the control plot was similar to that aforementioned, and five biological duplicate soil samples were obtained. The obtained samples were screened for impurities. One part was placed in a sealed bag for high-throughput sequencing. The other part was air-dried and preserved to determine soil physicochemical properties. In addition, number of branches (NOB), fresh weight (FW), internode length (ILH), plant height (PHT), and stem diameter (SD) were measured for 50 alfalfa plants (in each experimental plot).

**Table 4:** Basic information of alfalfa cultivars.

Code	Varieries name	Fall-Dormancy	Source	Dormancy type
0	CK	**/**	**/**	**/**
1	WL 712	10.0	America	Non (High) dormancy
2	WL 525HQ	8.0	America	
3	Victoria	6.0	America	Moderate dormancy
4	Kangsai	4.0	America	
5	Knight 2	2.0	America	Low dormancy
6	Aohan	1.0	China	

### Determination of soil physicochemical properties and phenotypic traits

Soil and water were mixed at a ratio of 1:2.5. Soil pH was measured by using a pH acidity meter (Model PHS-3C pH Meter, China). AN and SOM contents were determined using alkali solution diffusion and potassium dichromate oxidation-oil bath heating, respectively. We used 0.5 mol/L sodium bicarbonate as the extractant. The AP content was determined using molybdenum antimony colorimetry. Using 1 mol/L NH_4_OAc as the extractant, AK content in the soil was detected using the flame photometric method. The TN in the soil was determined using the Kjeldahl method. TP in the soil was determined using the HCIO_4_-H_2_SO_4_ digestion method. The TK in the soil was determined using sodium hydroxide melting and flame photometry. The detection and calculation of plant agronomic traits (PHT, FW, ILH, SD, and NOB) were based on a report in the literature^58^.

### High-throughput sequencing

Total DNA was obtained from the soil samples by using a soil DNA extraction kit (E.Z.N.A.® Soil DNA Kit, USA). PCR (ABI GeneAmp® 9700, USA) chain reactions were used to amplify these genes. Primers targeting the V3–V4 regions of 16s rRNA (338F: ACTCCTACGGGAGGCAGCAG; 806R: GGACTACHVGGGTWTCTAAT) containing barcodes were used^59^. Primers targeting the ITS1F-ITS2R regions (ITS1F: CTTGGTCATTTAGAGGAAGTAA; ITS2R: GCTGCGTTCTTCATCGATGC) containing barcodes were used. The amplified products were detected using 2% agarose gel electrophoresis. Next, we used a DNA gel recovery kit to recover the target band (Axygen Biosciences, USA) and verified this result by using 2% gel electrophoresis again. PCR products were quantified by a Quantus™ Fluorometer (Promega, USA), and the samples were adjusted for sequencing according to the required sequencing amount of each sample. Sequencing was performed by Shanghai Majorbio Biopharm Technology (Shanghai, China), which used the Illumina MiSeq platform.

### Sequence bioinformatics analysis

According to the overlapping relationship between PE reads, FLASH software (https://ccb.jhu.edu/software/FLASH/index.shtml) was used to merge pairs of reads into a sequence. The quality of the reads and the effects of merging were filtered using quality control. Sequences shorter than 200 bp, sequences containing N bases, and reads with an average mass of less than 20 were removed. Effective sequences were obtained by distinguishing the samples based on the barcode and primer sequences at both ends of the sequence, and the direction of the sequence was corrected. USEARCH v7.1 software was used to eliminate chimeras^60^. Operational taxonomic units (OTUs) of sequences with a similarity of 97% were determined using UPARSE software (version 7.1 http://www.drive5.com/uparse/)^61,62^. Rarefaction and Shannon curves were generated using OTUs. The SILVA and UNITE databases were used to classify the sequences of bacteria and fungi, respectively^63,64^. Relative abundance was calculated as the percentage of the sequence number of microbial taxa in the total sequence number of the samples.

### Statistical analysis

One-way ANOVA in SPSS software (version 26; Chicago, USA) was used to compare samples. MOTHUR software was used to calculate the diversity indexes of the microbial community (version v.1.30.2 https://mothur.org/wiki/calculators/). Based on the OTU Bray–Curtis algorithm, non-metric multidimensional scaling (NMDS) analysis was conducted using Vegan software to evaluate changes in the microbial community composition. Changes in the relative microbial abundance are represented by histograms and heatmaps. Redundancy analysis (RDA) and variation distribution were used to analyze the relationship between influential factors and the microbial community structure. Redundant variables that depended on other measurement variables were removed using RDA, and variables with a strong influence were selected automatically. The redundant parameters were removed from the variance expansion factor values. The LDA (LDA ≥ 3.5) of the groups of samples was performed using LefSe to identify meaningful organisms with significant differences. A heatmap was constructed using the R vegan software package (version 3.3.1). Spearman correlation analysis of the microbial community structure and environmental factors was performedusing *SPSS* software.

### Ethical approval

We confirm that the use of plants in the present study complies with international, national and/or institutional guidelines.

### Supplementary Information

Supplementary Figure S1.

Supplementary Figure S2.

Supplementary Figure S3.

Supplementary Figure S4.

Supplementary Table S1.

Supplementary Table S2.

Supplementary Table S3.

Supplementary Table S4.

Supplementary Table S5.

Supplementary Table S6.

Supplementary Table S7.

Supplementary Table S8.

Supplementary Table S9.

Supplementary Table S10.

Supplementary Table S11.

Supplementary Table S12.

## Data Availability

The data is available at the Sequence Read Archive (SRA) of NCBI: https://www.ncbi.nlm.nih.gov/sra/?term=PRJNA893894 (bacteria) and https://www.ncbi.nlm.nih.gov/sra/?term=PRJNA894156 (fungi).
