# Supplementary figures and images for: The effect of alfalfa cultivation on improving physicochemical properties soil microorganisms community structure of grey desert soil
Source: Sci Rep. 2023 Aug 23;13:13747. doi: 10.1038/s41598-023-41005-8 (PMC10447519; doi:10.1038/s41598-023-41005-8)

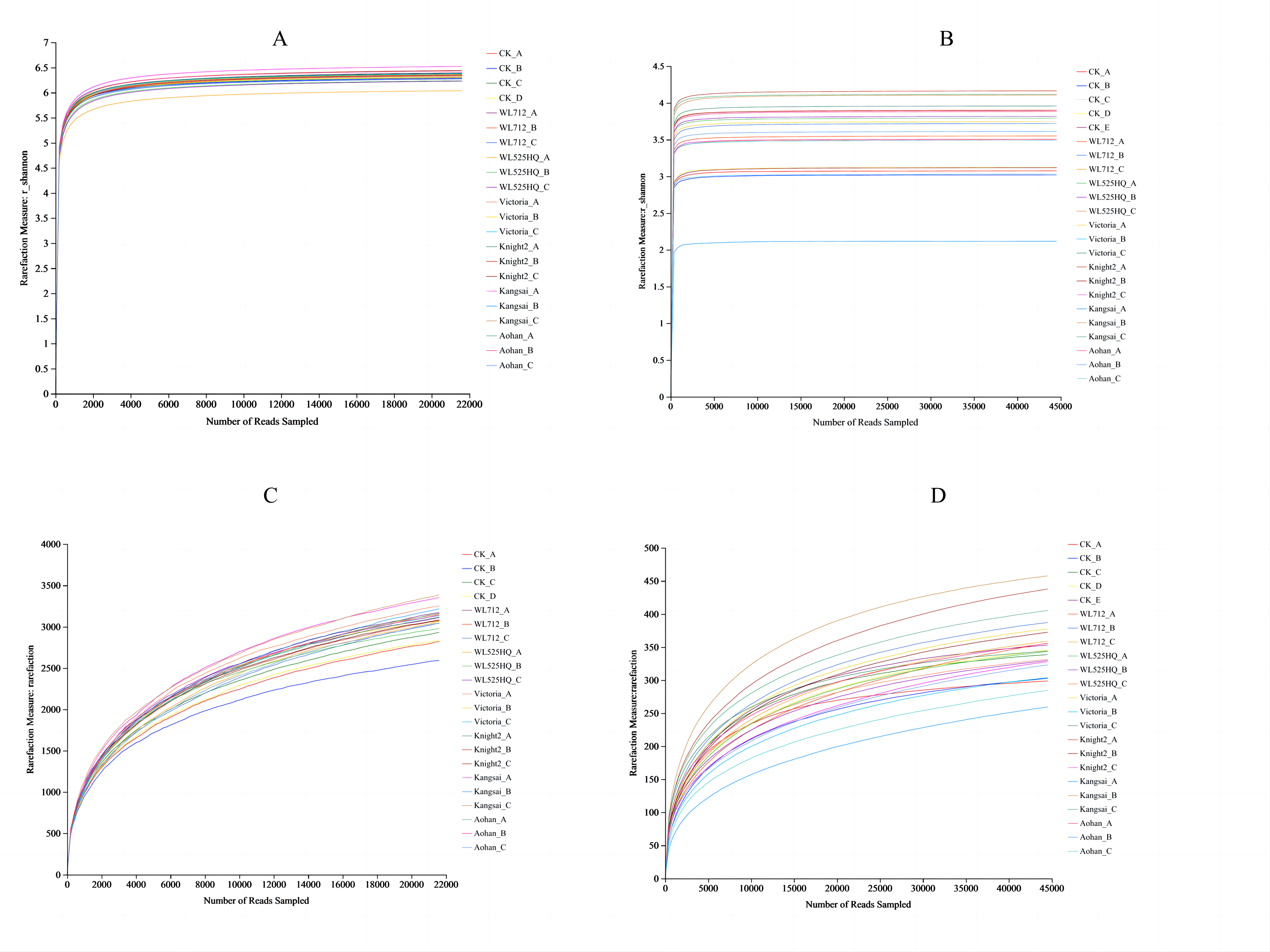

Supplement: Supplementary file 1 — Supplementary Figure S1. [file 41598_2023_41005_MOESM1_ESM.png]

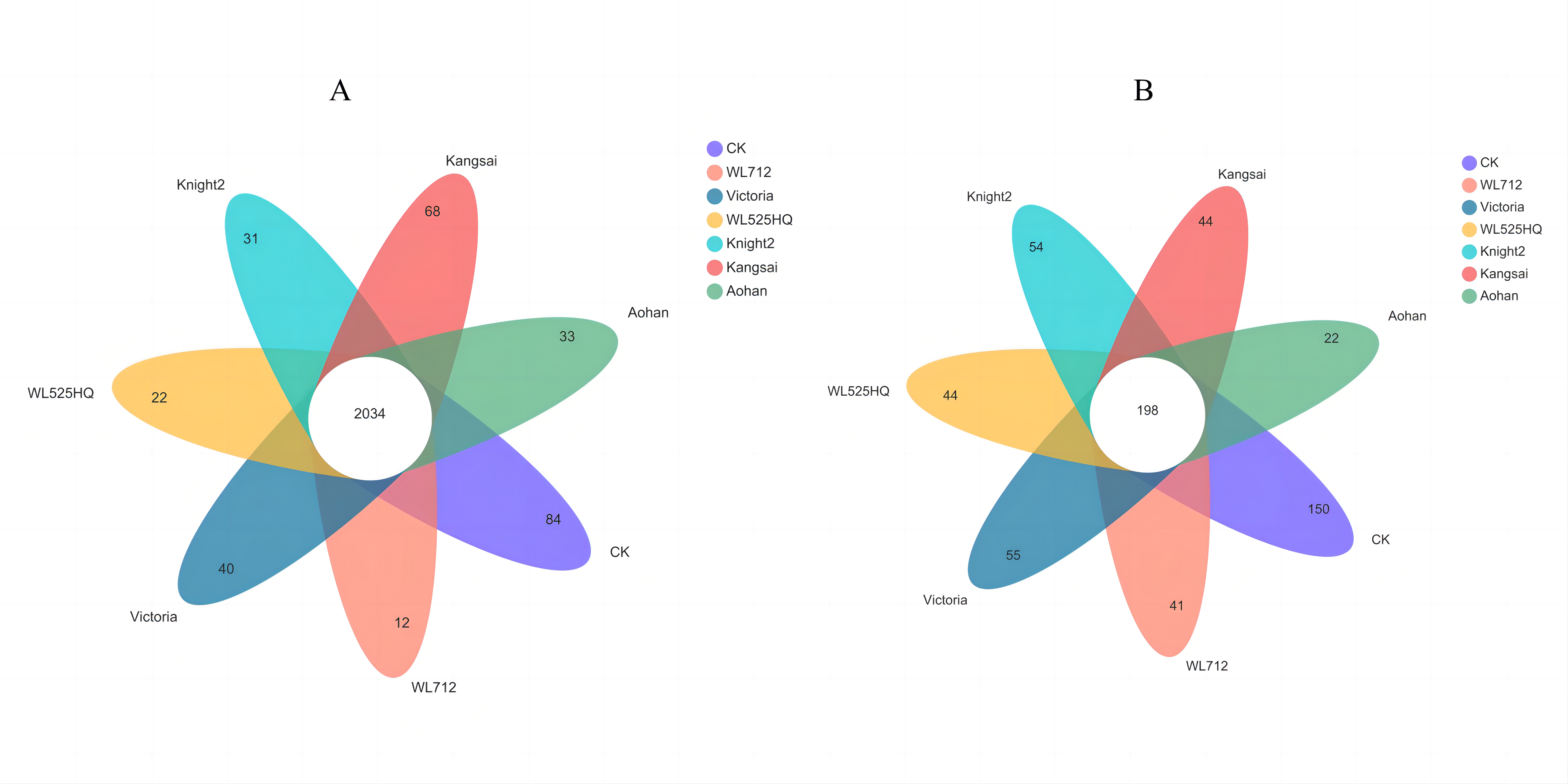

Supplement: Supplementary file 2 — Supplementary Figure S2. [file 41598_2023_41005_MOESM2_ESM.png]

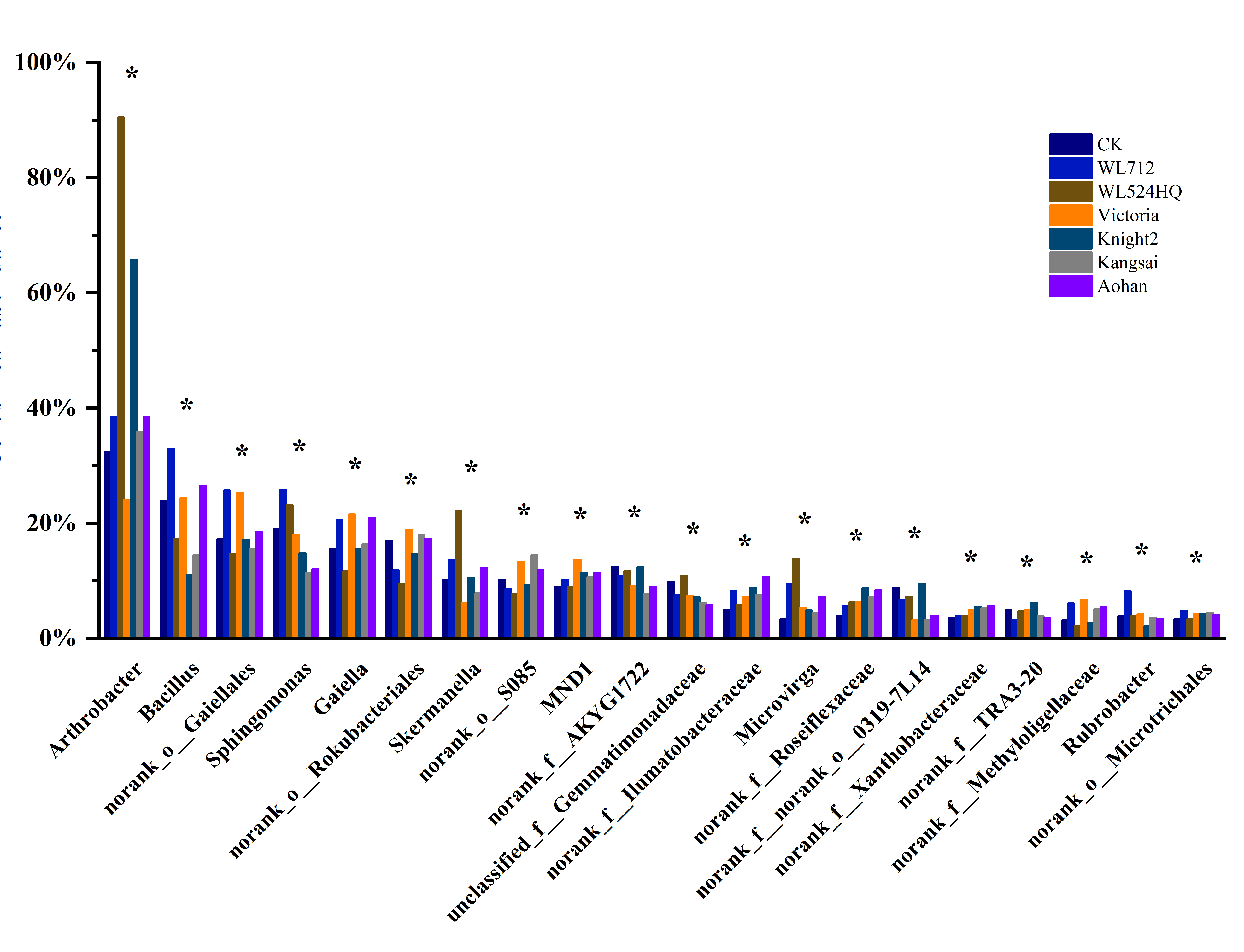

Supplement: Supplementary file 3 — Supplementary Figure S3. [file 41598_2023_41005_MOESM3_ESM.png]

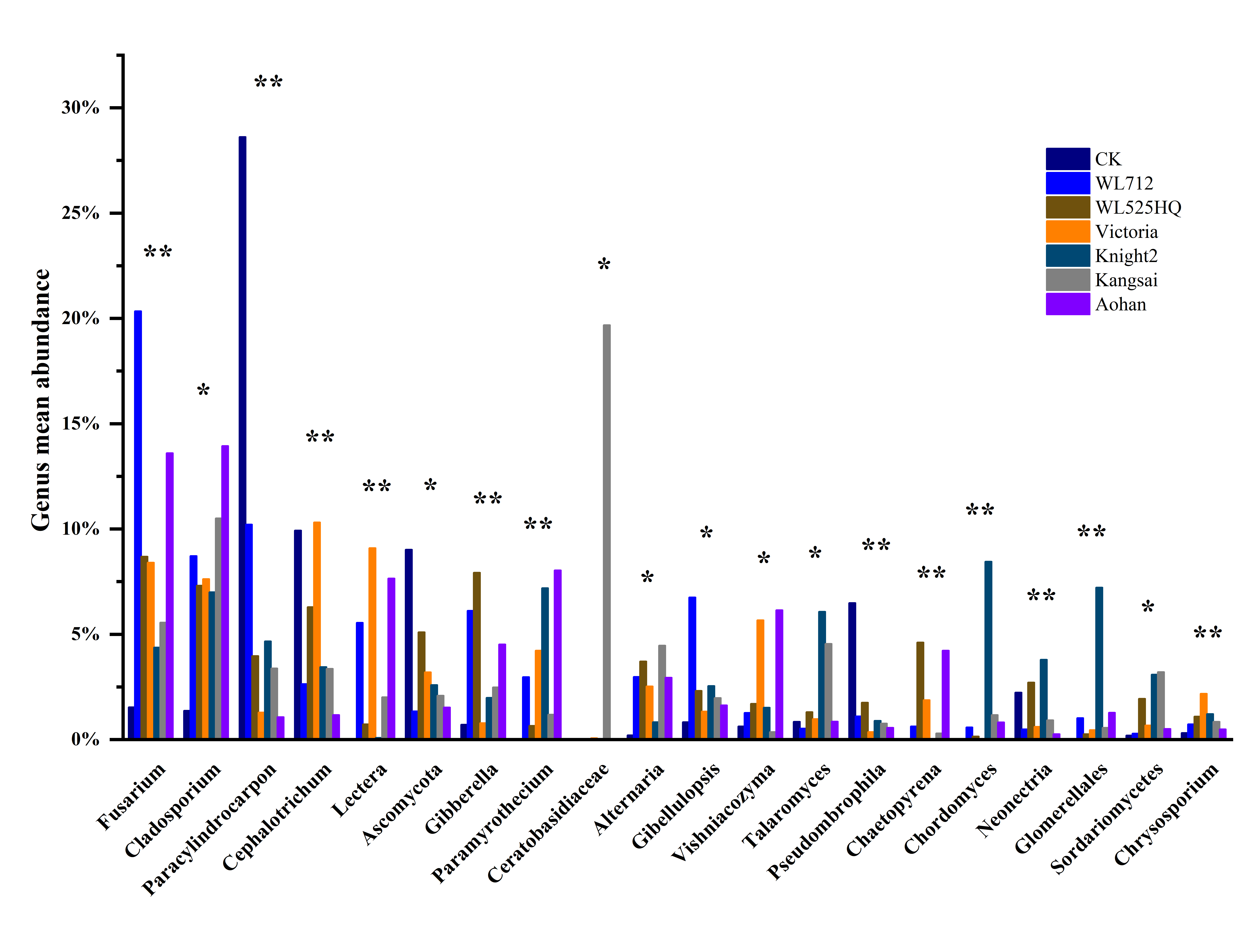

Supplement: Supplementary file 4 — Supplementary Figure S4. [file 41598_2023_41005_MOESM4_ESM.png]
